# The politics of non-communicable diseases in the global South

**DOI:** 10.1016/j.healthplace.2015.09.001

**Published:** 2016-05

**Authors:** David Reubi, Clare Herrick, Tim Brown

**Affiliations:** a Social Science, Health and Medicine, King's College London, United Kingdom; b Geography, King's College London, United Kingdom; c Geography, Queen Mary University of London, United Kingdom

**Keywords:** Non-communicable disease, Global South, Global health, Politics, Care

## Abstract

In this paper, we explore the emergence of non-communicable diseases (NCDs) as an object of political concern in and for countries of the global South. While epidemiologists and public health practitioners and scholars have long expressed concern with the changing global distribution of the burden of NCDs, it is only in more recent years that the aetiology, politics and consequences of these shifts have become an object of critical social scientific enquiry. These shifts mark the starting point for this special issue on ‘The Politics of NCDs in the Global South’ and act as the basis for new, critical interventions in how we understand NCDs. In this paper, we aim not only to introduce and contextualise the six contributions that form this special issue, but also to identify and explore three themes – problematisation, care and culture – that index the main areas of analytical and empirical concern that have motivated analyses of NCDs in the global South and are central to critical engagement with their political contours.

## 1 Introduction

Over the last 10 years, concern has been mounting over rapid rises in the prevalence of non-communicable diseases (NCDs) in the global South and the health and economic burden they represent. This has been driven, in part, by the World Health Organisation (WHO) which has published a number of reports on the topic and most recently adopted a *Global Action Plan for the Prevention and Control of Noncommunicable Diseases 2013–2020* (WHO, 2010, WHO, 2013). The World Bank and the United Nations Development Programme (UNDP) – two of the leading organisations in international development – have also been active and issued discussion and policy papers about ‘the mounting danger of chronic diseases’ for emerging economies (World Bank, 2011, UNDP, 2013). Governments, too, have expressed their alarm over this rising threat, recently passing a *Political Declaration on the Prevention and Control of Non-Communicable Diseases* at a high-level meeting of the United Nations' General Assembly, the second of only two such meetings held about health (United Nations, 2011). Of course, this attention to chronic diseases in the global South has not been the sole preserve of international organisations and governments. Public health and medical experts have long called for more attention to be paid to NCDs in this region. For example, one of the leading voices in the global health community, *The Lancet*, has published regular special issues with research on the epidemiological, economic and clinical aspects of chronic diseases since 2005 (e.g. Horton, 2005; Beaglehole and Horton, 2010; Geneau et al., 2010). Likewise, civil society and the private sector are showing a growing interest in the subject. Most significantly, in 2010, over two thousand health charities and patient organisations including the American Cancer Society and the World Heart Federation established, with support from the pharmaceutical industry, the NCD Alliance to lobby for and make chronic diseases a global health and development priority (Heath, 2011).

As these different actors have repeatedly argued, NCDs – defined in this context as comprising four conditions (cardiovascular disease, cancer, diabetes and chronic respiratory disorders) overwhelmingly caused by four behavioural risk factors (diet, physical activity, smoking and alcohol) – have become a critical issue for low and middle income countries (LMICs). Drawing on sophisticated epidemiological data, they point out that more than 60% of deaths worldwide are NCD-related and nearly 80% of these deaths occur in LMICs (WHO, 2010, UNDP, 2013). Indeed, in most countries across South America and Asia, chronic diseases are now the leading cause of death. Only in the African region are there more deaths from infectious diseases and even that is predicted to change over the next 15 years. This high prevalence of NCDs across the global South, these actors argue, constitutes ‘one of the major challenges for development in the 21st century’ (United Nations, 2011, p.1). As they explain, the relationship between chronic diseases and development is two-fold (World Bank, 2011, Alleyne et al., 2013, UNDP, 2013). On the one hand, the growing prevalence of NCDs in emerging economies is viewed as a negative consequence of socio-economic development, with economic growth and rapid urbanisation associated with a rise in ‘modern’ lifestyles (drinking, smoking, unhealthy diets, and physical inactivity) and an ageing population. On the other hand, the chronic disease epidemic in the global South is understood to be a serious threat to the sustainability of development through both its negative impact on the productivity of working age populations and the double burden of disease it places on health systems already overstretched by infectious, maternal and perinatal diseases. Predictably perhaps, many of the solutions put forward by these actors are health strategies successfully used in North America and Europe and which are deemed commensurate with the economic context of LMICs (Yach et al., 2006, Lim et al., 2007, Alwan et al., 2010, WHO, 2013). They include tools such as epidemiological surveillance systems as well as public health and clinical interventions that are ‘highly cost-effective cheap, feasible and culturally acceptable’ such as tobacco taxation, media campaigns for healthy diets and multidrug regimens for people at risk of cardiovascular diseases (WHO, 2010, p.47).

There has been no lack of academic attention given to the issue of NCDs in the global South from the public health community (Alleyne et al., 2011, Clark, 2014, Marrero et al., 2012, Stuckler and Basu, 2013). In contrast, critical social science engagements are comparatively rare, although interesting work has recently begun to emerge. For example, political scientists have examined the reasons behind the relative neglect of NCDs in global health policy and funding compared to issues like AIDS, pointing to the expert and advocacy networks involved and the ways issues are framed (e.g. Magnusson, 2007; Jönsson, 2014; cf. also Shiffman, 2009). Similarly, historians and others have started to explore the globalisation of chronic diseases and particular public health strategies like tobacco taxes over the last 50 years (e.g. Brown and Bell, 2008; Reubi, 2013; Weisz, 2014b). Another important part of this emerging body of work is the research carried out by anthropologists and geographers into the way ideas and practices associated with NCDs have been translated, resisted and re-appropriated when travelling to the global South. To illustrate, Livingston, 2012, Livingston, 2013 has pointed to the absence of pain relief medication and the very different understandings of pain in cancer wards in Botswana; while Lawhon and Herrick (2013; cf. also Herrick, 2013) have shown how alcohol control policies in Cape Town have been recast as an instrument to fight criminality rather than improve health. Others have looked into how the ideas and practices associated with NCDs have transformed subjectivities and notions of patienthood in the global South (e.g. Bunkenborg, 2003; Whyte, 2013; Whitmarsh, 2013; cf. also Whyte, 2012).

While this emerging body of critical studies on NCDs in the global South is a step in the right direction, much more needs to be done before we can start making sense of current initiatives to problematise and govern the chronic disease epidemic in emerging economies. So, for example, while the role of expert networks and discursive framings in problematising NCDs in the global South needs to be further scrutinised, we also need to explore the technologies and materialities like epidemiological maps and models that make it possible to view chronic diseases as a development issue. Likewise, while the influence of the tobacco, alcohol and food companies in globalising risk factors associated with NCDs is at risk of being over-analysed (e.g. Yach and Bettcher, 2000; Stuckler and Siegel, 2011), we know very little about the role of the pharmaceutical industry and philanthropic foundations in creating new markets for vaccines and drugs to treat chronic diseases in the region (e.g. Wailoo et al., 2010; Towghi, 2013; cf. also Petryna et al., 2006). It would also be helpful to know more about the complex relationships that exist between current initiatives to tackle NCDs and ideas and traditions that have been critical to the field of health and medicine such as post-colonialism, neoliberalism and securitisation (Collier and Lakoff, 2008, Elbe, 2010, Anderson, 2014). Last but not least, despite the efforts of some anthropologists (e.g. Livingston, 2005, Livingston, 2008), we still understand very little about the impact of NCD-related interventions on existing inequalities and the everyday lives of the poor in the global South (Farmer, 2005). More generally, then, there is a need to know more about the types of places that produce chronic diseases in the global South and, in turn, the ways in which the politics of NCDs reform and reshape places and people in the name of risk management and disease control.

The contributions in this special issue are an attempt to begin addressing these and other similar questions and themes. To locate these contributions within the broader critical social science literature on global health (e.g. Collier and Lakoff, 2008; Elbe, 2010; Weir and Mykhalovskiy, 2010; Stuckler and Siegel, 2011; Fassin, 2012; Farmer et al., 2013; Biehl and Petryna, 2013; Anderson, 2014), this introduction outlines three themes that, we believe, are central to a critical engagement with the politics of NCDs in the global South. First, an attention to ‘problematisation’ is an opportunity of examine and reflect on the conditions – intellectual categories, moral principles, geopolitical models, medical practices and other techniques – that make it possible to think about chronic diseases as a problem for developing countries today. Second, a concern with ‘care’ can help analyse and question contemporary NCD policies in the global South and their consequences by drawing on a tradition of social justice and human rights. Third, a focus on ‘culture’ offers a grid of analysis to explore and make sense of how both unhealthy lifestyles and public health policies associated with NCDs are translated, resisted and transformed when they travel from the global North to the global South. The six papers in this special issue represent important contributions to these themes while also highlighting the incredibly broad range, depth and complexity of concerns that the politics of NCDs invoke. These concerns then signpost future research agendas across the social sciences that are attuned to the current tenor of policy debates as well as shifts in the landscape of global health funding and programmatic priorities that will be drawn out at the end of this introduction.

## 2 The problematisation of chronic disease in the global South

For the most part, public health experts are concerned with confirming that NCDs are an issue for LMICs and articulating possible solutions. In contrast, more critically minded researchers are interested in exploring the conceptual, political and material conditions that make it possible to identify and think about chronic diseases as a problem of development today. Such an approach draws on the notion of ‘problematisation’ developed by Foucault, 1984, Foucault, 1988 and others (e.g. Rabinow and Rose, 2003; Hacking, 2002; Miller and Rose, 2008; Koopman, 2013). For these scholars, ‘problems are not pre-given, laying there waiting to be revealed’, but ‘have to be constructed and made visible’ through ‘a complex and often slow process’ (Miller and Rose, 2008, p.14). It is this ‘process of problematisation’ that they are keen to analyse in order to understand ‘how and why certain things (behaviour, phenomena, processes) became a problem’ (Foucault, 1984, p.17). This means exploring the ‘ensemble of discursive and non-discursive practices that make something enter into the play of true and false and constitute it as an object of thought, whether in the form of moral reflection, scientific knowledge or political analysis’ (Foucault cited in Rabinow and Rose, 2003, p.xviii-xix). Put differently, it involves studying the progressive development and assemblage of the scientific, moral and political rationalities, institutions, practices and techniques that make it possible to think of certain things as a problem today.

Taking such an approach, then, is to argue that chronic diseases in the global South are not a pre-given reality waiting to be revealed through ever more sophisticated epidemiological investigations but a problem that has been made thinkable through the progressive articulation of a complex assemblage of geopolitical categories, modernisation theories, biomedical practices and international networks of experts in health and development (Ong and Collier, 2008). We sketch here a tentative genealogy of some of these rationalities, practices and networks that make it possible to conceive of chronic diseases as a problem of development today. An important, early moment in such a genealogy has to be the elaboration of the notion of ‘health development’ in the post-World War II period (Walt and Rifkin, 1990). This was a period marked by the dismantling of the old colonial empires and the birth of the ‘Third World’ articulated through the theories and practices of development (Escobar, 1995). At first, the new development experts did not attach much importance to health. Indeed, for them, development was about economic growth and physical capital like roads, railways and industries. It is only from the 1960s onwards that they began to recognise that development was also about poverty alleviation and human capital. For the most part, this meant investing in education and healthcare systems so as to improve the quality and quantity of the labour force and bolster national productivity (Finnemore, 1997).

Another, critical step in the framing of chronic disease as a development issue was the articulation of the concept of chronic disease itself. As Armstrong (2014) has argued, this concept only came to prominence in the postwar period (cf. also Weisz, 2014a). The elaboration of this concept made it possible to bring together and view disorders such as cancer and heart ailments – which until then had been thought to be the product of the natural process of ageing and, as such, outside the realm of medicine – as part of a new diagnostic category: diseases with an aetiology of multiple, lifestyle-related risk factors that had a lasting impact on someone's capacity to function normally. As Armstrong (1995) also shows, this new diagnostic category came together with a new model of medicine – surveillance medicine – that progressively displaced pathological medicine from the 1950s onwards. Pathological medicine was about investigating the physiological lesion in the body of the patient in the hospital through clinical examinations, laboratory analyses and post-mortems (Foucault, 1976). In contrast, surveillance medicine was concerned with identifying possible risk factors of future illness through regular medico-social surveys and screening programmes of everyone in the community, both the ill and the seemingly healthy. Unlike pathological medicine, it also assumed a responsible patient who actively engaged in his or her surveillance, education and care, which comprised healthy lifestyles promotion campaigns, screening tests and life-long drug regimens (Petersen and Lupton, 2000).

For most public health experts, chronic diseases and the developing world were long thought to be mutually exclusive, with chronic diseases deemed to be the preserve of the rich, industrialised countries of the North while the major concern for the South was infectious diseases and malnutrition (Bryant, 1969, Brockington, 1985). In the minds of these experts, these differences in disease patterns were closely related with the demographic and socio-economic changes associated with modernisation. Perhaps the most influential account of this relationship between disease and modernity was Abdel Omran's notion of epidemiological transition. In Omran's terms, so-called ‘developed countries’ had undergone an epidemiological transition and entered the ‘Age of Degenerative and Man-Made Diseases’, which was not only characterised by chronic diseases but also by: low fertility, high life expectancy and ageing populations; economies articulated around technology and mass consumption; as well as rationality, nuclear families and high living standards (Omran, 1971, p.516–517). In contrast, ‘undeveloped’ societies, he posited, had yet to complete this transition and were still in the ‘Age of Pestilence and Famine’ defined not only by infectious diseases and malnutrition but also by: high fertility, high mortality and young populations; economies mixing subsistence farming with early industrialisation; as well as traditional values, extended families and poor, unsanitary living conditions (ibid.).

These different disease patterns and development levels were further associated with differing healthcare systems. While surveillance medicine was the dominant paradigm in North America and Europe, the notion of primary health care (PHC) enshrined in the Declaration of Alma Ata prevailed across the Third World (Fassin, 2000; Cueto, 2004). After independence, developing countries quickly realised that the healthcare systems inherited from colonial times and based around the hospital and eradication campaigns against tropical diseases were not appropriate to their situation: hospitals, usually located in cities, were not accessible to the rural poor that made up most of their population; eradication campaigns were associated with authoritarian practices that jarred with the spirit of decolonisation; and Western medical technologies were too expensive. PHC was developed as an alternative model of healthcare tailored to the specific needs of the Third World. It promised to offer essential healthcare made accessible to all citizens via a network of rural health workers and centres and characterised by community participation, an emphasis on prevention and simple, cheap technologies. While the programmes put in place to operationalise the PHC ideal varied across the developing world, they tended to concentrate on communicable diseases and child and maternal health issues, including: oral rehydration therapy for diarhoea; family planning; nutrition; and mass immunisations against major infectious diseases like measles and diphtheria (Mull, 1990).

This way of thinking, which deemed chronic diseases and the developing world as mutually exclusive and associated the latter with infectious diseases, maternal and child health, malnutrition and PHC remained predominant until the turn of the century. The Millennium Development Goals, for example, owed a lot to this style of reasoning, not least by viewing health as critical to development and by constraining its health-related efforts to maternal and child health, infectious diseases and malnutrition. But, from the late 1970s onwards, an increasing number of reports from physicians and mostly small, hospital-based epidemiological surveys in LMICs showing a growth in the number of patients suffering from NCDs began to challenge this way of thinking (Phillips, 1990, Reubi, 2013). Unsurprisingly, this gradually led to efforts to construe chronic diseases as a development issue. Of course, the WHO did some work on chronic diseases in the Third World, launching its Integrated Programme for Community Health in Non-Communicable Diseases in a small number of developing countries in the 1980s (Weisz, 2014a). But, it was the efforts of economists and epidemiologists at the World Bank – especially Dean Jamison's Health Sector Priorities Review, Richard Feachem's work on the Health of Adults in the Developing World and Christopher Murray's Global Burden of Disease Project – that would prove to be the most influential in identifying chronic disease as an issue for the global South and reconfiguring the relationship between development levels, disease patterns and healthcare models (Feachem et al., 1992, Jamison et al., 1993, Murray and Lopez, 1996).

There were many reasons for why the Bank's efforts proved to be so influential. First, this was a time when the Bank's investment in health-related projects grew exponentially, making it the world's premier health institution and pushing the WHO to the sidelines (Brown et al., 2006, Chorev, 2012). Second, the Bank's experts articulated a new understanding of the relation between development and disease that made it possible to think NCDs as an issue for LMICs. They suggested that one should stop classifying all developing countries together and recognise instead their growing economic and epidemiological diversity (Frenk et al., 1989, Jamison and Mosley, 1991). Specifically, complexifying Omran's model, they recommended distinguishing between two groups of developing countries: (i) low-income, usually African or South Asian, countries typified by infectious diseases and malnutrition; and (ii) middle-income, mostly East Asian or Latin American, countries characterised by a double burden of both infectious and chronic diseases (Jamison and Mosley, 1991). It was this second group – whose emergence was due to the success of existing PHC programmes at reducing infant mortality and changing patterns of risk such as unhealthy lifestyles generated by rapid urbanisation and rising incomes – that was the novelty and allowed the Bank's specialists to associate developing countries and chronic diseases for the first time (Bobadilla et al., 1993, Jamison et al., 1993, Mosley et al., 1993). Third, the claims about changing patterns of disease and development made by the Bank’s experts seemed to be supported by the new, allegedly more rigorous estimates of worldwide mortality and morbidity generated by Murray’s Global Burden of Disease project, something which was critical at a time when evidenced-based approaches were becoming all the rage (Murray and Lopez, 1996; Reubi, this issue). Fourth, the Bank's experts ensured that the problem of NCDs in the global South gained traction by linking it with a question that came to dominate the political agenda in most developing countries after the energy crises and global recession of the 1970s: how to finance healthcare systems in the face of mounting national debts and budgetary restrictions? (Rowden, 2009, Reubi, 2013). They did so through the notion of double burden of disease burden characteristic of the new, second group of developing countries, arguing that it would substantively add to the financial strain already impacting these countries’ healthcare systems (Frenk et al., 1989, Jamison et al., 1993). Fifth, unlike the WHO, the Bank was not wedded to PHC and was able to outline alternative healthcare models (Chorev, 2012). In particular, it argued that PHC programmes, with their focus on rural populations, infectious diseases and child and maternal health, had become too limited and called for a new healthcare model articulated around rational policies, epidemiological surveillance, cost-effective interventions focused on prevention and, sometimes, privatisation (Mosley et al., 1990, Birdsall and James, 1993).

Over the last fifteen years, the Bank has shown less interest in chronic disease and development, leaving the WHO and other organisations like the NCD Alliance and *The Lancet* to take the lead in this field (Weisz, 2014b). As mentioned at the start of this introduction, the numerous reports, action plans and scientific papers published by these organisations have further consolidated and propagated the ideas of NCDs as a development issue. Of course, these organisations have brought some of their own concepts and idiosyncrasies – like the WHO's addition of a reworked and weakened notion of PHC – to the way they frame this issue. But, overall, the way they conceive chronic diseases in the global South is strongly influenced by the analyses and ideas articulated by the World Bank's experts during the 1980s and 1990s. To illustrate, most of the documents on the topic published by these organisations share the Bank's understanding that the relationship between NCDs and development is a two-way process, with economic growth generating unhealthy lifestyles and reducing chronic disease prevalence critical to improving productivity (e.g. WHO, 2010; UNDP, 2013). Likewise, most of these documents, echoing the Bank, express the significance of the NCD epidemic in the global South through rigorous epidemiological data and emphasise the importance of using cost-effective health interventions and public-private partnerships (e.g. Lim et al., 2007; WHO, 2013).

## 3 Chronic diseases and the politics of care

A focus on problematisation is, of course, not the only critical approach that can be used to make sense of current efforts to tackle NCDs in the global South. Another, important lens through which to explore these efforts is a critique characterised by a concern with social justice and human rights (Benatar et al., 2003, Benatar, 2005, Kleinman, 2010, Venkatapuram, 2010). This frame points to the political importance of care to the ways in which we approach NCDs across a number of domains. Specifically, the invocation of social justice and human rights acts as a critique of current approaches to NCDs in two ways. First, of the global health community's selective deployment of the tools, techniques, funds and interventions that permits the care of people. Second, of the ability of the state to ensure the adequate care of its citizens. If the first critique calls the contemporary architecture of global health into question (Farmer et al., 2013, Garrett, 2013), then the second scrutinises the ability of this architecture to deliver sustainable, effective and equitable health improvements on the ground (Benatar, 2005, Venkatapuram, 2010). The politics of NCDs in the global South are thus bound into and directly shaped by the nature, delivery and critique of care by a variety of actors. The ability and will to care, in turn, is shaped by the complex, multi-scalar politics and resource flows that condition so much of the global health enterprise. Care implies a need for empathy, responsibility and duty just as much as it does the fair distribution of medical services and resources and the capacity to access and make use of these (Kearns and Reid-Henry, 2009). It is therefore an essential – if under-acknowledged – component of the politics of NCDs in countries of the global South.

The capacity to care is constrained by a number of factors that warrant further scrutiny. In the first place, current efforts to address chronic diseases in LMICs are indubitably limited by the very delineation of NCDs themselves. This, in turn, draws attention to the ways in which the global health enterprise is so often enacted within a number of specific and siloed realms, with little structural capacity to deal with the implications of the complex porosity of definitional categories. For example, ‘the boundaries between communicable and non-communicable diseases are often indistinct’ (Farmer et al., 2013, p. 321). So, it could be argued that, with the development of antiretroviral therapies, AIDS has become a chronic disease that can be managed through life-long drug regimens and changes in one's lifestyle. Similarly, some have argued that cervical cancer, a current priority of the global health community, is more akin to a communicable disease given that it is triggered by the sexually-transmitted Human Papilloma Virus (HPV) and is now preventable through a vaccine (Livingston, 2012). There are, moreover, similar boundary problems with mental health issues like depression that are excluded from the official NCD definition but yet seem to fit the notion of a disease that has a lasting impact on someone's capacity to function in society (WHO, 2010). The notion of NCD also partakes in the ‘mistake of pitting one set of pathologies against another’ for attention and funding from the global health community, instead of promoting an approach to public health policy and practice that is intersectoral and holistic (Farmer et al., 2013, p.322). Interestingly, this division and fragmentation is also encouraged by the focus on discrete, cost-effective health interventions developed by the World Bank at the end of the 20th century and taken over by the WHO and others over the last 15 years.

Another issue relates to the capacity of the state to provide adequate care for its citizens and, moreover, the consequences of this for the sustainability of global health programmes (Marmot et al., 2008). Failure in this domain is both deflected and reinforced by the lack of focus on the social determinants of NCDs and the role of mounting inequalities in entrenching these. Moreover, the twin phenomena of globalisation (Beaglehole and Yach, 2003, Yach and Beaglehole, 2004) and rapid urbanisation (Mitlin and Satterthwaite, 2012, WHO/UN-HABITAT, 2009) have unsettled the assumptions inherent within the epidemiological transition model. Now, households are gripped not just by the ‘double burden’ of disease (Bygbjerg, 2012), but in some cases, a ‘triple’ or even ‘quadruple burden’ that also includes injuries and violence, as well as perinatal and maternal diseases (Bradshaw et al., 2003). Crucially, the characteristics of these burdens vary not just between countries, but also within them and at ever-finer geographic scales. Even within one household, for example, there might be underweight, malnourished family members living alongside equally malnourished obese relatives (Doak et al., 2004). It is important then to consider the social determinants of this complicated and multi-layered disease burden: poverty, inequality, quality of housing, access to sanitation, unemployment, education, transport, food security, the nature of healthcare provision and environmental degradation. It is these structural, economic, political and social drivers that largely condition the dynamics of the four main risk factors for chronic disease: diet, exercise, alcohol and tobacco (WHO, 2005). Yet, while the proportion of people living in poverty may have fallen (United Nations, 2013), rates of both inequality and, perhaps even more importantly, inequity, within many countries is accelerating (OECD, 2011). This means that while advances in medical science remain essential to reducing mortality and morbidity, there is also an absolute imperative for ‘economic and social policies that would improve basic living conditions’ for all household members in LMICs (Benatar et al., 2003, p. 110). Moreover, it must also be acknowledged that while urbanisation creates new behavioural risks for those living in cities, in many LMICs, it also produces a profound care gap in which older family members are left in rural areas without either adequate health infrastructure or family networks to care for them in times of illness (Livingston, 2003). These transitions, in turn, test the capacity of the state just as much as the current machinery of global health.

With its overwhelming focus on single diseases and technocratic solutions, global health does offer a model of care, but it is one that can often be problematically short-lived and partial (Garrett, 2007). It can also be outcomes rather than process-orientated. This raises the question of the type of care global health endeavours to provide and for whom. Indeed, the degree to which the mechanisms of global health penetrate broader social structures and, as a result, the determinants of health, is a question that is infrequently asked and nowhere near being solved. Under conditions where global health activities have supplanted the responsibilities of the state, there is the danger that this may start to precipitate ‘a striking culture of indifference to affliction present in areas of extreme inequality’, which, in turn, ‘facilitates a pathogenic biosocial spiral of socioeconomic exclusion and deteriorating health’ (Nguyen and Peschard, 2003, 448; see also Farmer, 2005; Quesada et al., 2011). Thus, while many countries of the global South have witnessed meteoric climbs in their middle classes, the gulf between rich and poor has only widened. Across the global South, there are also mounting inequities between state and private healthcare provision, the distribution of essential medical technologies, drugs and expertise as medical professionals seek employment in the global North, the cities of Asia and the Gulf (Mills et al., 2011; Parry et al., 2015). This necessarily means a situation where ‘the rich, although increasingly shielded from most disease threats, are able to purchase better health’ (Nguyen and Peschard, 2003, 449) and may actually only rarely come into direct contact with global health programmes. Moreover, when the richest can access healthcare elsewhere, this does little to either inculcate a broader ethic of care or to bolster support for efforts to address the wider social determinants of health (Hall, 2011). As a result, social justice and human rights remain a persistent absence in the politics of NCDs in the global South.

This absence is further reinforced by the fracturing of the social solidarities that have traditionally underpinned an ethic of care in the face of global change. This in turn reveals a further, painful irony at work in efforts to tackle NCDs in the global South. NCDs require not only the care of others, but also necessitate care of the self, especially in relation to the four major lifestyle risk factors. This need is occurring just as the traditional state-centred mechanisms of care and the will to care may be being eroded, not least because of the reforms associated with structural adjustment policies (Rowden, 2009). NCDs require adherence to both prevention and treatment regimes. Both are amenable to some degree of individual control (such as not smoking or drinking in moderation), but are equally often determined by the structural factors underpinning the distal pathogenic effects of inequality. These can erode real choices as well as the capacity to make reasoned choices. Further complicating care, treatments for NCDs, even if available, may be expensive or their supply intermittent. Treatment regimes may require lifetime adherence (e.g. statins for high cholesterol), certain levels of competence (e.g. diabetes blood sugar testing and insulin therapy), expensive technologies or complex surgical techniques (e.g. MRI scanners, laser surgery) or basic palliative medications such as analgesics that are unavailable (Beaglehole et al., 2011, 1442). Moreover, where infectious disease and NCDs coexist, as they so often do in the global South, existing poor health, compromised immunity or episodic illness may undermine the capacity to undertake either prevention or treatment activities. Not only may this ignite conditions under which the rhetoric of individual blame may be invoked, but it also ensures, as Livingston, 2005, Livingston, 2008, Livingston, 2012 exemplary work in Botswana has explored, that people need more care, often earlier in their lives and the consequences of illness can be catastrophic in terms of economic and social disenfranchisement.

## 4 Chronic diseases and the politics of culture

Another, third way to critically examine the politics of NCDs in the global South is through the lens of ‘culture’. This immediately begs the question: what is meant here by culture? Do we mean culture in the normative sense, as a ‘thing’ (for example, a set of health-related practices, beliefs or behaviours) that is shared by a specific cultural group or within a geographical space that is argued to have dominant cultural norms (Dutta, 2008)? It is certainly the case that such a normative perspective has been mobilised in analyses of non-communicable diseases in the global South as elsewhere; especially those that highlight the importance of lifestyle risk factors. Such analyses are often framed by a different transition model to the ones we have already discussed; to the epidemiologic and health transition models we can add the idea of the nutrition transition (Popkin, 1994). This latter model emphasises the relationship between levels of economic growth or development and patterns of dietary behaviour. As Drewnowski and Popkin (1997) explain, diets at one time primarily associated with the rich industrialised nations of the global North – the so-called ‘western’ diet, which is high in fats, especially meat and milk products, saturated fats and sugars – are no longer regarded as being spatially fixed. Put simply, relatively early studies into the structure of global diets in the 1960s and 1970s suggested that as GNP per capita rises within nations so too does the consumption of foods associated with the western diet. More recent analyses, such as that offered by Drewnowski and Popkin, 1997, Pingali, 2007, Kearney, 2010, add further layers of understanding to this fairly simplistic model by suggesting that a host of other factors, including urbanisation, global food advertising and marketing and associated shifts in socio-cultural practice, also play an important role in this transition (Hawkes, 2006).

Of particular concern here is the question of the rapidity of the nutrition transition and the importance of culture to it; as Chopra et al., (2002, p. 954; cf. also: WHO, 2002; WHO/FAO, 2003) remarked, ‘[a]larm has been expressed about the rapid spread of the fast food culture, perhaps exemplified most visibly by McDonald's’. There is a tendency in analyses that draw on culture in this way to treat it as a ‘thing’ that invades or colonises other, often by implication indigenous or ‘traditional’, cultural practices; as Uusitalo et al. (2005, p. 608) explain, ‘the diffusion and adoption of Western culture in other places is often termed “Westernisation”, whereby societies and individuals adopt particular ideas and practices from more economically developed and commercialised countries’. So, for example, studies such as theirs point to the replacing of ‘indigenous’ foods with ‘western’ ones: rice, fish and vegetables for eggs, dairy and meat. This surprisingly imperialist vision of an invasive western culture mirrors long-standing concern with the impact of acculturation on the food habits of migrants; as shown, for example, in relatively early studies of migration, dietary change and chronic heart disease in 1960s USA (cf. Syme et al. 1965; Marmot and Syme, 1976).

Such analyses of cultural transition, here relating to dietary behaviours, highlight the disruptive tendencies of social change brought about by processes such as rapid urbanisation or the globalisation of cultural practices and their often negative influence on population health (Szreter, 1999). This is certainly an important area for further academic enquiry especially in the many and diverse countries that make up the global South. Indeed, as Whyte (2012) notes, most ‘cross-cultural’ studies of this kind have been carried out in the multi-ethnic settings of high-income countries in the global North. However, it is not the only way in which we might approach the question of culture as it relates to the politics of NCDs. Crawford's (1984) influential essay on cultural approaches to health is useful here. As he argues, the body is a ‘cultural object’; one that provides a ‘powerful medium through which we interpret and give expression to our individual and social experience’ (Crawford, 1984, p. 60; see also Lupton, 2012). Bodies are differentially constituted as healthy, diseased, risky and so on across a range of media and with consequences that are felt at different spatial scales as well as at the level of individual bodies. While this is perhaps especially so with regards infectious or contagious bodies, all bodies that are understood as out of control or outside of socially constituted notions of normality – obese ones, depressed ones, cancerous ones, psychotic ones, intoxicated ones – are those upon which political and ideological optics are focused (Craddock and Brown, 2009, Brown et al., 2012).

A further illustration of the importance of engaging critically with the politics of culture and health that emerge here comes from the discourse of the contemporary obesity epidemic. Across the social sciences, there is a lively and often contentious debate relating to Foucault's concepts of biopower and biopolitics and how they might be drawn upon in critical analyses of non-communicable diseases in general and obesity more specifically (e.g. Wright and Harwood, 2009; Lupton, 2013). The participants in this debate not only contest the science of obesity (e.g. Gard and Wright, 2005; Guthman, 2011) but, more importantly here, they critique the pathologisation of fatness and an associated governmental impulse that prioritises the production of bodies that conform to cultural norms regarding size and shape as well as to contemporary public health imperatives relating to individual and population health (e.g. Evans, 2006; Evans and Colls, 2009; Fullagar, 2009; Herrick, 2011). As Bethan Evans and Rachel Colls argue, such an impulse is *biopolitical* in that individuals are the subjects of ‘surveillance, punishment and training’ and relates to Foucault's broader understanding of *biopower* because the discourse surrounding obesity is directed at ‘man-as-species’ and is concerned more generally with the health of populations (Foucault cited in Evans and Colls, 2009, p. 1055).

Evans (2010) extends this reading of the biopolitics of obesity in a subsequent essay discussing notions of threat and pre-emptive politics as they relate to the body, the population and the nation. As she argues, the ‘war’ on obesity that has developed in the high-income countries of the global North, which is a war on specific types of bodies as much as it is a war on the environments that help to produce them (Guthman, 2011), is concerned primarily with the threat that the ‘*matter* of bodies’ pose in the future (Evans, 2010, p. 22). Evans distinguishes here between the public health logics of *prevention* and *pre-emption* and focuses on interventions directed at the ‘bodies of the future’: children (Evans, 2010, 30). Her argument is that where the western tradition of public health has in the past concerned itself with the prevention of known and calculable risks to health, it is now more focused with taking pre-emptive action in the face of futures that are less certain, less knowable. As she argues, obesity policy is ‘reliant on the temporal gap between onset of risk factor and onset (or not) of ill-health. This gap provides an opportunity for pre-emptive action…’ (Evans, 2010, p. 30).

There are two key points to take from the above discussion. Firstly, if we only treat culture normatively in our analyses of NCDs in the global South, as there has been a tendency to do, we risk obscuring the political contestation that arises around specific bodies and the (western) practices that have rendered them problematic. Yach et al. (2006) suggest there is a threat inherent in the importation of western medical responses; for them, it relates to the pharmaceuticalisation of public health as well as to the reliance on procedures such as bariatric surgery (cf. Whitmarsh, 2013). Arguing from a health economics perspective and for more emphasis on evidence-based prevention strategies, they suggest such interventions risk the vitality of entire health systems as money is diverted to expensive and unaffordable treatments. We would argue this is not the only ‘threat’ that needs critical attention. To it we would add the threat posed by neoliberal ideologies that have underpinned the response to NCDs in the global North and which see care for certain bodies not only as an ‘excessive cost’ in the present but as an unacceptable burden on the future (Guthman, 2011, 54). Secondly, and more briefly, the above discussion challenges us to consider more seriously and much more critically the emerging preventive and pre-emptive strategies that are being put in place in the global South to assure against the apparent threat posed by western cultural practices.

## 5 An outline of the special issue

The six papers of this special issue help shed light, in varying ways, on our respective concerns with problematisation, care and culture within the politics of NCDs in the global South. Reubi's (this issue) paper explores how epidemiological models used to problematise smoking in developing countries are building on notions of time and space associated with postwar theories about modernisation and progress. In this reading – favoured by tobacco control activists – development and, by extension, the kinds of “globalised” culture that are presumed to be the hallmark of economic growth become proxies for epidemiological risk. Here, development and culture are constructed as specific and serious threats to public health, with political intervention the favoured solution. Criticism of the simplistic readings of culture that can dominate the politics of NCDs is also a feature of Smit et al.'s (this issue) paper in which they explore the recursive relationships between the built environment and the experience of chronic disease in the context of Khayelitsha, one of Cape Town's poorest neighbourhoods. Their paper highlights two glaring absences in the problem frames of the global NCD agenda – mental health and the entanglements of urban environments with upstream determinants of health. The gross inequalities in everyday life within cities not only condition the likelihood of suffering from chronic disease, but also the shape and nature of that suffering. Cultural coping mechanisms, in turn, can be severely compromised by the nature of places and their use. Smit et al. draw attention to the problems of food purchasing and storage, of being physically active and of the depression and stress that emerge from living with the perpetual (fear of) crime and violence. Coping, Smit et al. argue, will only be enhanced through attention to the drivers of risky environments, issues that remain silent in a politics of NCDs that would prefer to blame the failings of culture than acknowledge the complicity of the state in producing risk.

This critique is also a feature of Glasgow and Schrecker's (this issue) paper in which they argue that the political imaginaries of global health, shaped as they are by inherently neoliberal ideologies, purposefully divert attention from both the social and political economic determinants of NCDs. Instead, they place responsibility for NCDs and their prevention in the hands of individuals, rendering care a matter of successful cultural behavioural interventions. The concern with individual choice, responsibility and empowerment also represents the hope that new, self-governing subjects can be formed that, in turn, can exercise a culture of self-care. Such a culture is essential for the success of most contemporary NCD prevention and treatment strategies, yet so much critical social scientific analysis demonstrates just how problematic these political aspirations are. For example, MacDonald's (this issue), Bunkenborg's (this issue) and Whyte's (this issue) papers all engage with the politics of NCDs through the experiences of largely ‘improvised’ (Livingston, 2012) treatment options for breast cancer and diabetes available in India, China and Uganda respectively. These papers speak directly to each other in their concern with the new forms of biosociality and therapeutic citizenship that arise in the need to care for those suffering with chronic diseases in places where the medical treatment provided by public hospitals, charities and private clinics is either insufficient or at odds with local cultural models of health and illness. For MacDonald and Whyte, this care takes the form of expert patients and patient groups that help mediate the often inadequate relationship between doctor and patient and provides the empathy and information needed to plug gaps in the existing provision of care. For Bunkenborg, these new forms of sociality and citizenship do not only involve expert patients and patient groups but are also mediated through the commercial world of diabetes treatments and technologies. In his example of China, the doctor-patient relationship is often fraught with mistrust, providing an opportunity for a cacophony of private enterprises to bring the hope of diabetes self-management through a range of products and drugs. In each of these examples, the experience of NCDs, the cultural formations that emerge from them and the demand for care are inextricable from the complex and desperately uneven public-private patchwork of medical services. Inadequate care is rarely a matter of cultural failing, despite the blame tendencies of individualised behavioural framings of NCDs. In understanding the interweaving of politics, care and culture in the problematisation of NCDs in the global South it is hoped that this collection of papers will open up new conversations about these issues and help us think how politics and policies might be reshaped in ways that enhance their ability to alleviate human suffering.
